# Whole genome sequences of 234 indigenous African chickens from Ethiopia

**DOI:** 10.1038/s41597-022-01129-4

**Published:** 2022-02-14

**Authors:** Almas Gheyas, Adriana Vallejo-Trujillo, Adebabay Kebede, Tadelle Dessie, Olivier Hanotte, Jacqueline Smith

**Affiliations:** 1 Centre for Tropical Livestock Genetics and Health (CTLGH), The Roslin Institute, University of Edinburgh, Midlothian, EH25 9RG UK; 2 Cells, Organism and Molecular Genetics, School of Life Sciences, University of Nottingham, Nottingham, NG7 2TQ UK; 3 LiveGene – CTLGH, International Livestock Research Institute (ILRI), P.O. Box 5689, Addis Ababa, Ethiopia; 4 Amhara Regional Agricultural Research Institute, Andassa Livestock Research Centre, P.O. Box 27, Bahir Dar, Ethiopia

**Keywords:** Next-generation sequencing, Genetic markers, Genomics, Agricultural genetics

## Abstract

Indigenous chickens predominate poultry production in Africa. Although preferred for backyard farming because of their adaptability to harsh tropical environments, these populations suffer from relatively low productivity compared to commercial lines. Genome analyses can unravel the genetic potential of improvement of these birds for both production and resilience traits for the benefit of African poultry farming systems. Here we report whole-genome sequences of 234 indigenous chickens from 24 Ethiopian populations distributed under diverse agro-climatic conditions. The data represents over eight terabytes of paired-end sequences from the Ilumina HiSeqX platform with an average coverage of about 57X. Almost 99% of the sequence reads could be mapped against the chicken reference genome (GRCg6a), confirming the high quality of the data. Variant calling detected around 15 million SNPs, of which about 86% are known variants (i.e., present in public databases), providing further confidence on the data quality. The dataset provides an excellent resource for investigating genetic diversity and local environmental adaptations with important implications for breed improvement and conservation purposes.

## Abstract (summary)

**Table:** 

Measurement(s)	genome
Technology Type(s)	DNA sequencing
Factor Type(s)	animal population
Sample Characteristic - Organism	Gallus gallus
Sample Characteristic - Location	Ethiopia

Machine-accessible metadata file describing the reported data: 10.6084/m9.figshare.16999891

## Background & Summary

Poultry farming constitutes an important economic activity across Africa, providing a livelihood for millions of people. However, the lion’s share of the poultry production in most countries still comes from smallholder backyard indigenous poultry reared under scavenging or semi-scavenging conditions, with no or limited human intervention (e.g., secured sheltering at night, supplementary feeding, or vaccination)^1,2^. Ethiopia is one of the sub-Saharan African countries where chicken farming plays a crucial role in the country’s sociocultural context and economy, with ~97% of the production still coming from “extensive” farming practice of local birds.

Domestic chickens were originally introduced into Ethiopia from Asia from around 3000 years ago^3,4^. Since their introduction, chicken populations have been dispersed throughout the country and, over time, have adapted to thrive in its diverse agro-ecologies. These birds, now considered as indigenous, show greater resistance to various local poultry diseases and parasites compared to exotic and commercially improved chickens. Due to their superior adaptability to local tropical environmental conditions as well as their foraging ability and broodiness, these indigenous birds are often preferred by smallholder farmers for backyard rearing^2,5,6^. However, in the absence of proper management practices or any systematic selection efforts, local birds generally show poorer productivity but higher survivability compared to the commercial counterparts. Their untapped genetic potential can be utilized for improving their performance.

Genome analyses can unravel the genetic diversity of indigenous chicken populations and provide the basis for genetic improvements for better production and performance. Moreover, genome analysis of populations from different agro-ecological zones can elucidate the genetic basis of local environmental adaptation. Resilient genotypes, identified from such studies, can then be selected for or introgressed in improved productive breeds for superior performance under local climate. The Ethiopian landscape can be considered a microcosm of different agro-ecologies encountered in Africa due to extreme variations in its altitudinal topography and rainfall pattern. This has given rise to diverse agro-climate zones in the country, ranging from hot-arid and hot-humid to cold-humid and cold-arid^7^. Therefore, genomic analysis of Ethiopian chicken populations is particularly pertinent for elucidating their local adaptation.

**Fig. 1:**
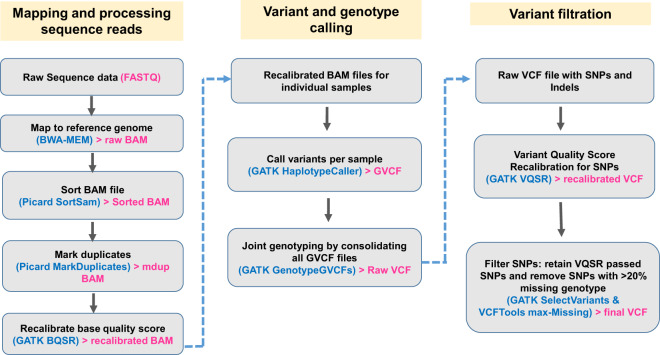
Overview of the sequence mapping, variant calling and variant filtration pipeline. The pipeline follows GATK best practice protocol for germline short variant discovery^18^.

This article reports whole genome sequencing data from hundreds of indigenous chickens (n = 234), sampled from 24 different Ethiopian villages or populations distributed under diverse agro-ecological and climatic conditions [Table 1; also see Fig. 1A,B and supplementary Table S1 in the study by Gheyas *et al*.^8^]. The study also reports about 15 million Single Nucleotide Polymorphisms (SNPs) detected by mapping the sequencing data against the chicken reference genome (*GRCg6a*; https://www.ncbi.nlm.nih.gov/assembly/?term=GCA_000002315.5). Sequencing has been performed at a very high coverage (average 57X), increasing the power and resolution of genomic analyses. Although most of the reported variants are already known (only 14% are novel), the associated VCF file (submitted to European Variant Archive) shows genotype data for individual samples; therefore it offers an excellent resource for a variety of population genetics analyses. Some of these sequences and variant data have been used in a recent study to elucidate the genome-environmental adaptation in Ethiopian chickens^8^.

**Table 1:** Details of Ethiopian chicken populations.

Population IDs as appear in ENA database	No. of samples	Geographic region	District	Village or Kebele
Afar;Dulecha;Hugub	10	Afar	Dulecha	Hugub
Afar;Dulecha;Kefis	10	Afar	Dulecha	Kefis
Amhara;Banja;Surta	9	Amhara	Banja	Surta
Amhara;FagitaLekoma;AmeshaShinkuri	10	Amhara	Fagita Lekoma	Amesha Shinkuri
Amhara;FagitaLekoma;Batambie	8	Amhara	Fagita Lekoma	Batambie
Amhara;FagitaLekoma;Gafera	10	Amhara	Fagita Lekoma	Gafera
Amhara;GondarZuria;TsionTeguaz	10	Amhara	Gondar Zuria	TsionTeguaz
Amhara;Kalu;0–25Adane	10	Amhara	Kalu	0–25Adane
Amhara;Kalu;Arabo	10	Amhara	Kalu	Arabo
Amhara;MenzGeraMidir;AlfaMidir/05/	10	Amhara	Menz Gera Midir	Alfa Midir/05/
Amhara;MenzGeraMidir;NegasiAmba/07/	10	Amhara	Menz Gera Midir	Negasi Amba/07/
Amhara;SouthAchefer;Ashuda	10	Amhara	South Achefer	Ashuda
Amhara;SouthAchefer;Dikuli	10	Amhara	South Achefer	Dikuli
Gumuz;Dibate;Gesses	10	Gumuz	Dibate	Gesses
Gumuz;Dibate;Kido	9	Gumuz	Dibate	Kido
Oromia;Dugda;BekeleGirissa	10	Oromia	Dugda	Bekele Girissa
Oromia;Dugda;ShubiGemo	10	Oromia	Dugda	Shubi Gemo
SNNPR;Dara;Kumato	10	SNNPR	Dara	Kumato
SNNPR;Dara;Loya	10	SNNPR	Dara	Loya
Tigray;Enderta;Meseret	10	Tigray	Enderta	Meseret
Tigray;Merebleke;HadushAdi	9	Tigray	Merebleke	Hadush Adi
Tigray;Merebleke;Mihquan	10	Tigray	Merebleke	Mihquan
Tigray;SaharetiSamire;Gijet	9	Tigray	Sahareti Samire	Gijet
Tigray;SaharetiSamire;Metkilimat	10	Tigray	Sahareti Samire	Metkilimat

^$^Also see Supplementary Table S1 in Gheyas *et al*.^8^.

The data are expected to have many utilities, ranging from exploring genetic diversity, identifying signatures of positive selection, analysing genome-environment associations, finding genetic variants from regions of interests (e.g., within or near candidate genes or QTLs associated with disease and production traits), exploring different types of genetic variants (e.g., small insertions/deletions, structural variants, avian retroviral elements), and for developing tools for genomic analysis (e.g., high or low density SNP genotyping arrays for use in breeding programmes). Furthermore, the data represent the largest number of indigenous chicken samples sequenced from an African country. Only a few studies have previously reported such large scale sequencing of chicken samples but none generated such large scale African data^9–12^. These data are therefore a rich addition to global chicken genome sequence databases and can be used in conjunction with sequencing data from other countries/regions around the globe for studying demographic and domestication histories in chicken.

## Methods

### Chicken sampling

Chicken sampling considered different agro-climatic conditions and geographic regions of Ethiopia. Sampling of local foraging chickens was performed from 24 villages or ‘kebeles’ from across six regional states – Afar, Amhara, Gumuz, Oromia, SNNPR (Southern Nations, Nationalities and Peoples’ Region), and Tigray, representing diverse agro-climatic and ecological conditions observed in Ethiopia. Each village was considered as a separate population. To capture genetic diversity within populations, 8 to 10 chicken samples were collected from each village (Table 1). Sampling was performed by drawing blood (50–250 µl) from the wing vein of each bird with syringes using cryotubes filled with 1.5 ml absolute ethanol (100%) following the guidelines available at https://www.sheffield.ac.uk/nbaf-s/protocols_list. The samples consisted of 146 female and 88 male birds (total 234) and varied in their age (4–30 months; average 10.3 months) and body weight (0.6–2.6 kg, average 1.27 kg). The samples were collected with the logistical support and agreement of the Ethiopian Ministry of Agriculture and Ethiopian Institute of Agricultural Research (EIAR). All animal works were approved by the Institutional Animal Care and Use Committee of the International Livestock Research Institute (IREC2017-26). The sample information has been submitted to the European Nucleotide Archive (ENA) under the study accession *PRJEB39275*^13^ (see Online-only Table 1 for details about the samples).

### Genomic DNA isolation and quality control

All the collected blood samples were processed for DNA extraction at the BecA-ILRI Hub facility, Nairobi, Kenya (http://hub.africabiosciences.org/) using the Qiagen DNeasy blood and tissue kit protocol (https://www.qiagen.com/ca/resources/download.aspx?id=63e22fd7-6eed-4bcb-8097-7ec77bcd4de6&lang=en). DNA concentration was evaluated by spectrophotometry (Thermo Scientific NanoDrop spectrophotometer 2000c) and the integrity of DNA was confirmed by agarose gel electrophoresis. The genomic DNA (gDNA) from each sample was then normalized to a final volume of 100 µl and final concentration of 50 ng/µl and was sent to Edinburgh Genomics, UK for whole genome sequencing (WGS). At Edinburgh Genomics, gDNA samples were re-evaluated for quantity and quality using an AATI Fragment Analyzer and the DNF-487 Standard Sensitivity Genomic DNA Analysis Kit https://www.agilent.com/cs/library/usermanuals/public/quick-guide-dnf-487-genomic-dna-kit-SD-AT000137.pdf. The AATI ProSize 2.0 software (https://dna.biotech.iastate.edu/fragmentanalyzer.html) provided a quantification value and a quality (integrity) score for each gDNA sample. Samples with a score >7 passed quality control. Based on the quantification results, gDNA samples were pre-normalised to fall within the acceptable range for library preparation.

### Sequence library preparation and quality control

Next Generation sequencing libraries were prepared using Illumina SeqLab specific TruSeq Nano High Throughput Library preparation kits in conjunction with the Hamilton MicroLab STAR and Clarity LIMS X Edition. The normalized gDNA samples were sheared to a 450 bp mean insert size using a Covaris LE220 focused-ultrasonicator. The inserts were ligated with blunt ended, A-tailed, size selected TruSeq adapters and enriched using eight cycles of PCR amplification. The libraries were evaluated for mean peak size and quantity using the Caliper GX Touch with a HT DNA 1k/12 K/HI SENS LabChip and HT DNA HI SENS Reagent Kit. The libraries were normalised to 5 nM using the GX data and the actual concentration was established using a Roche LightCycler 480 and a Kapa Illumina Library Quantification kit and Standards (https://rochesequencingstore.com/wp-content/uploads/2017/10/KAPA-Lib-Quant-ILMN_9.17-IfU_1.pdf).

### Sequencing

The libraries were denatured, and pooled in groups of eight for clustering and sequencing using a Hamilton MicroLab STAR with Genologics Clarity LIMS X Edition. Libraries were clustered onto HiSeqX Flow cells v2.5 on cBot2s and the clustered flow cells were transferred to a HiSeqX for sequencing using a HiSeqX Ten Reagent kit v2.5. Sequencing was performed in paired-end mode with read length of 150 bp.

### Sequencing data processing, mapping and variant calling

Demultiplexing was performed using bcl2fastq (v2.17.1.14)^14^, allowing a single mismatch when assigning reads to barcodes. Adapters (Read1: AGATCGGAAGAGCACACGTCTGAACTCCAGTCA, Read2: AGATCGGAAGA GCGTCGTGTAGGGAAAGAGTGT) were trimmed during the demultiplexing process. Sequencing data quality was checked using the FASTQC package (v0.11.5)^15^. FASTQC reports for all samples were aggregated in a single report by the MultiQC package^16^ for easy review of sequence quality. No quality-based trimming was performed on the sequence reads prior to mapping and sequencing data from all samples were processed.

Sequence reads were mapped against the latest version of chicken reference genome (GCA_000002315.5_GRCg6a) using the BWA-mem (v0.7.15) algorithm^17^. The resulting SAM/BAM files from the mapping step underwent a series of further processing steps, including coordinate sorting (using the SortSam function in Picard v2.9.2), duplicate reads marking (using MarkDuplicates function in Picard) and Base Quality Score Recalibration (BQSR) using GTAK v3.8-0. The final recalibrated BAM files were then used for variant calling. Figure 1 shows an overview of the mapping and variant calling steps.

SNP calling was performed following the GATK best practice protocol for “Germline short variant discovery”^18^ using the HaplotypeCaller function on individual samples followed by joint genotyping (using GenotypeGVCFs function) of the samples. Variant filtration was performed by applying the Variant Quality Score Recalibration (VQSR) approach^19^ in GATK (v 3.8-0) using about one million validated SNPs^20^ as a training and true set, and over 20 M known chicken SNPs from the Ensembl database as known variants. During the VQSR step the following annotations or context statistics were considered: read depth (DP), variant quality by depth (QD), root mean square of mapping quality (MQ), mapping quality rank sum test statistic (MQRankSum), read position rank sum test statistic (ReadPosRankSum), and strand bias statistics (FS and SOR). A tranche sensitivity threshold of 99% was applied for filtering variants. The “Code availability” section below shows the specific codes for each mapping and variant-calling step. As the final quality control of the called variants, any SNPs with a missing genotype rate of more than 20% across the samples were filtered out using VCFtools (option – max-missing 0.8).

## Data Records

The raw full-length sequencing data (in FASTQ format) have been submitted to the European Nucleotide Archive (ENA) under the accession number *PRJEB39275*^13^. The VCF file of ~15 M SNPs detected from this dataset has been deposited in the European Variation Archive (EVA) with the accession number for Project: *PRJEB46494*^21^ and Analyses: *ERZ2899764*.

## Technical Validation

### Quality control of sequencing data

**Fig. 2:**
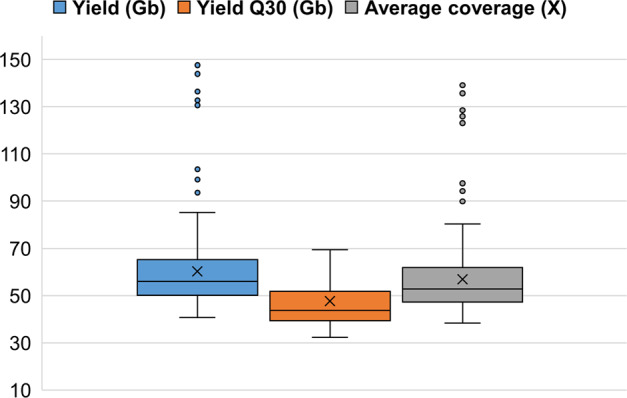
Boxplots showing the distributions of sequencing yield, yield Q30 and estimated coverage for Ethiopian chicken samples (n = 234).

**Fig. 3:**
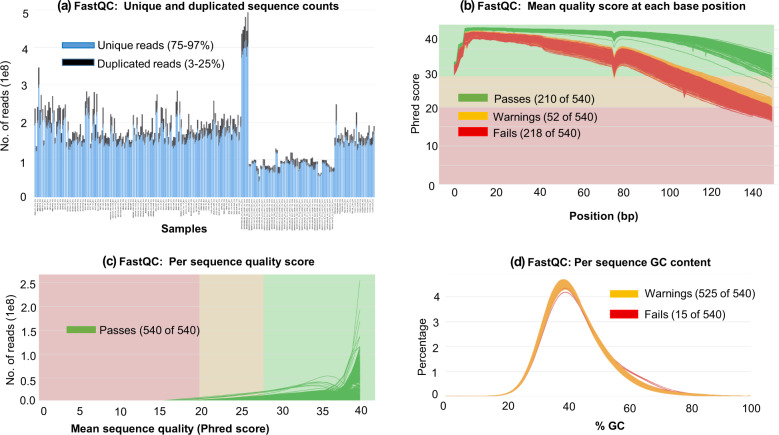
Quality control metrics from FastQC analysis of sequencing data. The metrics from all sequence FASTQ files (total 540) are combined using the MultiQC package.

For each sample, 41 Gb to 148 Gb sequencing yield (number of bases generated) was obtained, of which 74–83% of the bases (average 79%) had a minimum Phred scaled quality score of 30, indicating expected base calling accuracy of 99.9% (Fig. 2). The average estimated coverage for the samples varied from 38X to 139X (average across all samples 57X) (Fig. 2). Figure 3 shows selected features from FASTQC reports regarding sequencing quality (consolidated for all samples by the MultiQC package). This confirmed overall high quality sequencing data. Although Fig. 3b shows “Fails” signal for many reads, this should not be a matter of concern. All these “Fails” signals are associated with Read2 of the paired reads. Typically, Read2 often has a lower average quality than Read1^22^. A gradual drop in sequencing quality towards the end of the reads is also typical and expected of Illumina sequencing. It is important to note that Fig. 3d confirms a high average quality score for all reads. The mapping success rates of the sequence reads against the chicken reference genome were very high – 98.2% to 99.5% - which further confirmed the high quality of the sequencing data.

### Quality control of SNP data

Joint genotyping of all samples originally identified about 25 M SNPs. To ensure variant quality and minimize false positives, VQSR filtration was applied. By using machine learning algorithms, the VQSR method clusters the called variants based on annotation profiles of a set of known true positive SNPs (training set) in the detected set and calculates, for each variant, a new score called VQSLOD (https://gatk.broadinstitute.org/hc/en-us/articles/360035531612-Variant-Quality-Score-Recalibration-VQSR-). For filtration of the variants, we applied a VQSLOD threshold that retained 99% of the training variants. This filtration retained about 19 M SNPs. Further filtration based on missing genotypes (removed any SNPs with missing rate >20%) retained ~15 M good quality SNPs. About 86% of these variants have already been reported in the public databases. This provides extra confidence in the validity of the detected SNPs.

Transition and transversion ratio (Ti/Tv) is used as a quality control metric for SNP calling. For whole genome sequencing data, the typical value is ~2^23^. A higher ratio generally indicates better SNP calling unless the ratio is too high (>4)^24^. We obtained a Ti/Tv ratio of 2.38 for 19 M SNPs after VQSR filtration and a ratio of 2.5 for the 15 M final set.

**Fig. 4:**
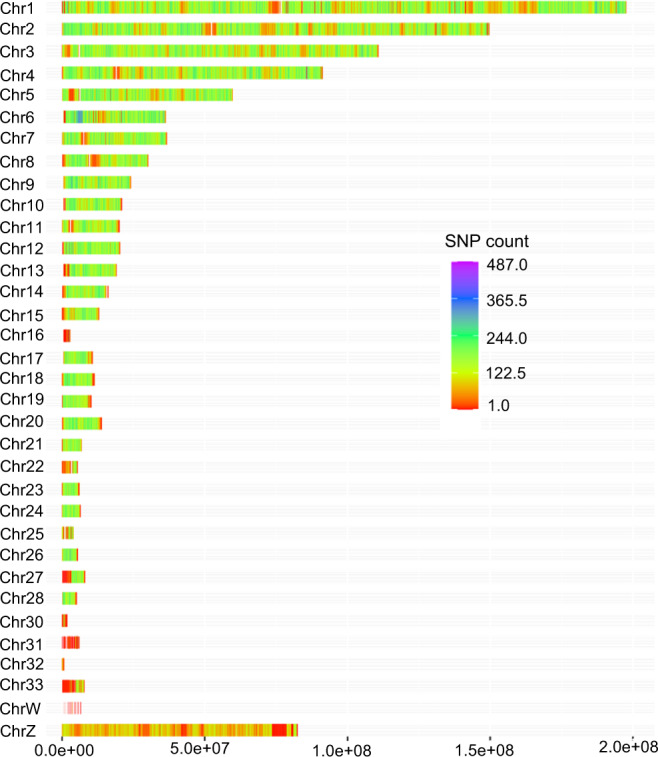
Chromosome-wise SNP distribution heat map across the Ethiopian indigenous chicken genomes based on 15 M SNPs. X-axis denotes the chromosome size in base pairs (bp) and Y-axis the chromosome number. The SNP count was calculated for 10 kb non-overlapping windows.

Table 2 and the heat maps of SNP density across different chromosomes in Fig. 4 show a good representation of most chromosomes and regions except some microchromosomes (e.g., chr16, 22, 25, 30–33) and the sex chromosomes (Fig. 4). Chromosome 16 is known to have a high repeat content^25^ whereas most microchromosomes have higher GC contents^26^; both causing difficulty in sequencing and mapping. The detected SNPs also had a good representation of different annotation categories in relation to their positions within or outside genes (Table 3).

**Table 2:** Summary statistics of SNPs in the VCF file for each chromosome.

Chromosome	GenBank accession of chromosome (as appears in the VCF)	SNP count	SNP density (count/kb)
1	CM000093.5	2,928,344	14.82
2	CM000094.5	2,239,989	14.96
3	CM000095.5	1,661,035	14.99
4	CM000096.5	1,417,213	15.52
5	CM000097.5	910,264	15.22
6	CM000098.5	620,260	17.05
7	CM000099.5	572,074	15.57
8	CM000100.5	424,726	14.05
9	CM000101.5	399,626	16.55
10	CM000102.5	314,978	14.91
11	CM000103.5	278,391	13.78
12	CM000104.5	329,825	16.18
13	CM000105.5	290,349	15.15
14	CM000106.5	249,997	15.41
15	CM000107.5	182,245	13.95
16	CM000108.5	7,904	2.78
17	CM000109.5	164,256	15.26
18	CM000110.5	184,132	16.19
19	CM000111.5	155,991	15.11
20	CM000112.5	219,725	15.81
21	CM000113.5	108,592	15.86
22	CM000114.5	38,943	7.13
23	CM000115.5	95,108	15.47
24	CM000116.5	105,193	16.21
25	CM000124.5	33,975	8.54
26	CM000117.5	93,980	15.52
27	CM000118.5	76,540	9.48
28	CM000119.5	77,753	15.20
30	CM003637.2	6,825	3.75
31	CM003638.2	8,658	1.40
32	CM000120.4	3,987	5.49
33	CM000123.5	35,838	4.59
W	CM000121.5	108	0.02
Z	CM000122.5	59,1904	7.17
unplaced	—	7,210	—

**Table 3:** SNPs in different annotation categories.

Annotation categories	count	% of total
exonic-nonsynonymous	63,008	0.425
exonic-synonymous	140,659	0.948
exonic-stopgain/loss	722	0.005
intronic	6,867,836	46.279
splicing	458	0.003
ncRNA_exonic	145,986	0.984
ncRNA_intronic	1,413,260	9.523
ncRNA_splicing	867	0.006
UTR3/UTR5	159,062	1.072
up/donwstream	501,901	3.382
intergenic	5,546,213	37.373
Total	14,839,972	

## Data Availability

Most of the data analyses were completed by standard bioinformatic tools running on the Linux system. The version and code/parameters of the main software tools are described below. **(1) BWA-mem (v0.7.15); code for mapping reads**: bwa mem -t 1 -M -R “@RG\tID:${SAMPLE}\tSM:${SAMPLE}\tPL:Illumina\tLB:${SAMPLE}\tPU:unkn-0.0” ${REF} ${READS_1} ${READS_2} > ${SAMPLE}.sam **(2) Picard (2.9.2): code for sorting sam file and converting to bam**: java -jar picard.jar SortSam I = ${SAMPLE}.sam O = ${SAMPLE}_sorted.bam SORT_ORDER = coordinate TMP_DIR = tmp_${SAMPLE} **(3) Picard (2.9.2): code for marking duplicate reads:** java -jar picard.jar MarkDuplicates I = ${SAMPLE}_sorted.bam O = ${SAMPLE}_mdup.bam CREATE_INDEX = true M = ${SAMPLE}_mdup_metrics.txt TMP_DIR = tmp_${SAMPLE} MAX_FILE_HANDLES_FOR_READ_ENDS_MAP = 4000 OPTICAL_DUPLICATE_PIXEL_DISTANCE = 2500 **(4) GATK (3.8-0): codes for BQSR steps** ***# Analyse patterns of covariation in the sequence dataset*** java -jar $gatk -T BaseRecalibrator -R ${REF} -I ${SAMPLE}_mdup.bam -knownSites ${KNOWNVAR} -o ${SAMPLE}_recal_data.table ***# Analyse covariation post-recalibration*** java -jar $gatk -T BaseRecalibrator -R ${REF} -I ${SAMPLE}_mdup.bam -knownSites ${KNOWNVAR} -BQSR ${SAMPLE}_recal_data.table -o ${SAMPLE}_post_recal_data.table ***# Generate before/after plots*** # Requires R packages gsalib, reshape and ggplot2 installed java -jar $gatk -T AnalyzeCovariates -R ${REF} -before ${SAMPLE}_recal_data.table -after ${SAMPLE}_post_recal_data.table -plots ${SAMPLE}_recalibration_plots.pdf ***# Apply the recalibration to your sequence data*** java -jar $gatk -T PrintReads -R ${REF} -I ${SAMPLE}_mdup.bam - BQSR ${SAMPLE}_recal_data.table -o ${SAMPLE}_recal.bam **(5) GATK (3.8-0) Variant calling in GVCF mode by HaplotypeCaller** java -jar $gatk -T HaplotypeCaller -R ${REF} -I ${SAMPLE}_recal.bam -o ${SAMPLE}.g.vcf.gz -ERC GVCF **(6) GATK (3.8-0) Joint genotyping of a cohort of samples** # used the --variant option as many times as needed to specify the gvcf files to be used for joint genotyping (the code below shows three samples only as example). java -Xmx4g -jar $gatk -T GenotypeGVCFs -R ${REF} --variant SAMPLE1.g.vcf.gz --variant SAMPLE2.g.vcf.gz --variant SAMPLE3.g.vcf.gz -o ${COHORT}.vcf.gz -D ${KNOWNVAR} **(7) GATK (3.8-0) VQSR steps** ***# Variant recalibration step*** java -Xmx4g -jar $gatk -T VariantRecalibrator -R ${REF} -input ${COHORT}.vcf.gz -resource:GRCg6a_dbsnp,known = true,training = false,truth = false,prior = 2.0 ${KNOWNVAR} -resource:GRCg6a_validated_snp,known = false,training = true,truth = true,prior = 12 ${TRUEVAR} -an DP -an QD -an MQ -an MQRankSum -an ReadPosRankSum -an FS -an SOR -mode SNP -tranche 100.0 -tranche 99.9 -tranche 99.0 -tranche 90.0 -recalFile ${COHORT}.SNPs.recal.gz -tranchesFile ${COHORT}.SNPs.tranches -rscriptFile ${COHORT}_recalSNPS.plots.R ***# Apply Recalibration*** java -Xmx4g -jar $gatk -T ApplyRecalibration -R ${REF} -input ${COHORT}.vcf.gz -mode SNP --ts_filter_level 99.0 -recalFile ${COHORT}.SNPs.recal.gz -tranchesFile ${COHORT}.SNPs.tranches -o ${COHORT}_recalSNPs_rawIndel.vcf.gz

## Online-only Table

**Online-only Table 1:** Ethiopian indigenous chicken samples analysed.

Sample ID	ENA study accession	ENA sample accession	Geographic region	District	Village or kebele	Read pairs sequenced	Yield (Gb)	Yield Q30 (Gb)	Average coverage (X)	Sex	Estimated age (month)	body weight (kg)
ABB-2H_Pink	PRJEB39275	SAMEA7050617	Amhara	Fagita Lekoma	Batambie	236903685	71	57	67	F	12	1.5
ABB-3C	PRJEB39275	SAMEA7050618	Amhara	Fagita Lekoma	Batambie	136048918	41	32	38	M	8	1.75
ABB-4H1	PRJEB39275	SAMEA7050619	Amhara	Fagita Lekoma	Batambie	241166137	72	58	68	F	7	1
ABB-4H2	PRJEB39275	SAMEA7050620	Amhara	Fagita Lekoma	Batambie	346459637	103	84	98	F	7	1
ABB-5H1	PRJEB39275	SAMEA7050621	Amhara	Fagita Lekoma	Batambie	233217499	70	56	66	F	12	1.8
ABB-6Cock1	PRJEB39275	SAMEA7050622	Amhara	Fagita Lekoma	Batambie	273287822	82	67	77	M	6	1
ABB-6H2	PRJEB39275	SAMEA7050623	Amhara	Fagita Lekoma	Batambie	235530885	70	58	66	F	12	1.8
ABB-6H3	PRJEB39275	SAMEA7050624	Amhara	Fagita Lekoma	Batambie	152188006	45	36	43	F	10	1
ABS-1C	PRJEB39275	SAMEA7050625	Amhara	Banja	Surta	245482239	73	59	69	M	8	1.5
ABS-1H	PRJEB39275	SAMEA7050626	Amhara	Banja	Surta	216663455	65	52	61	F	10	1.5
ABS-2H	PRJEB39275	SAMEA7050627	Amhara	Banja	Surta	231834678	69	55	65	F	7	1.5
ABS-3C	PRJEB39275	SAMEA7050628	Amhara	Banja	Surta	254625156	76	60	72	M	5	1.5
ABS-4C	PRJEB39275	SAMEA7050629	Amhara	Banja	Surta	239438827	72	57	67	M	6	1.8
ABS-5H1	PRJEB39275	SAMEA7050630	Amhara	Banja	Surta	198637437	59	47	56	F	12	1
ABS-5H2	PRJEB39275	SAMEA7050631	Amhara	Banja	Surta	270614377	81	65	76	F	14	1.5
ABS-6H	PRJEB39275	SAMEA7050632	Amhara	Banja	Surta	224653163	65	51	63	F	9	2
ABS-7H	PRJEB39275	SAMEA7050633	Amhara	Banja	Surta	231822222	69	56	65	F	8	2
AFA-1C	PRJEB39275	SAMEA7050634	Amhara	Fagita Lekoma	Amesha Shinkuri	159125452	48	37	45	M	12	1
AFA-1H	PRJEB39275	SAMEA7050635	Amhara	Fagita Lekoma	Amesha Shinkuri	202087813	60	49	57	F	13	1.5
AFA-2H	PRJEB39275	SAMEA7050636	Amhara	Fagita Lekoma	Amesha Shinkuri	237574115	71	57	67	F	12	1.5
AFA-3C	PRJEB39275	SAMEA7050637	Amhara	Fagita Lekoma	Amesha Shinkuri	246350017	74	60	69	M	5	1
AFA-4C	PRJEB39275	SAMEA7050638	Amhara	Fagita Lekoma	Amesha Shinkuri	239431575	72	57	67	M	6	1.5
AFA-4H	PRJEB39275	SAMEA7050639	Amhara	Fagita Lekoma	Amesha Shinkuri	170867009	51	40	48	F	12	1.5
AFA-5C	PRJEB39275	SAMEA7050640	Amhara	Fagita Lekoma	Amesha Shinkuri	235330808	70	56	66	M	7	1.5
AFA-6H	PRJEB39275	SAMEA7050641	Amhara	Fagita Lekoma	Amesha Shinkuri	238841700	71	58	67	F	12	1
AFA-7H	PRJEB39275	SAMEA7050642	Amhara	Fagita Lekoma	Amesha Shinkuri	248000703	74	61	70	F	8	1
AFA-8H	PRJEB39275	SAMEA7050643	Amhara	Fagita Lekoma	Amesha Shinkuri	147424380	44	35	42	F	7	1
AFDH-C1	PRJEB39275	SAMEA7050644	Afar	Dulecha	Hugub	202434489	61	47	57	M	24	1.4
AFDH-C3	PRJEB39275	SAMEA7050645	Afar	Dulecha	Hugub	156927338	47	37	44	M		1
AFDH-C5	PRJEB39275	SAMEA7050646	Afar	Dulecha	Hugub	155819772	47	37	44	F	8	0.9
AFDH-C7	PRJEB39275	SAMEA7050647	Afar	Dulecha	Hugub	194400157	58	46	55	M		
AFDH-H1	PRJEB39275	SAMEA7050648	Afar	Dulecha	Hugub	191026735	57	44	54	M		
AFDH-H2	PRJEB39275	SAMEA7050649	Afar	Dulecha	Hugub	151570048	45	35	43	F	12	1.6
AFDH-H4	PRJEB39275	SAMEA7050650	Afar	Dulecha	Hugub	217737906	64	49	61	F		
AFDH-H6	PRJEB39275	SAMEA7050651	Afar	Dulecha	Hugub	174428592	52	41	49	F	5	0.8
AFDH-H8	PRJEB39275	SAMEA7050652	Afar	Dulecha	Hugub	170865081	51	40	48	F	7	0.9
AFDH-H9	PRJEB39275	SAMEA7050653	Afar	Dulecha	Hugub	176562813	53	44	50	F	24	1.3
AFDK-C1	PRJEB39275	SAMEA7050654	Afar	Dulecha	Kefis	196898525	57	45	55	M		
AFDK-C2	PRJEB39275	SAMEA7050655	Afar	Dulecha	Kefis	481693349	144	113	136	M		1.4
AFDK-C3	PRJEB39275	SAMEA7050656	Afar	Dulecha	Kefis	444457131	133	101	125	M		1.05
AFDK-H10	PRJEB39275	SAMEA7050657	Afar	Dulecha	Kefis	173434225	52	42	49	F	12	0.97
AFDK-H4	PRJEB39275	SAMEA7050658	Afar	Dulecha	Kefis	160968698	48	38	45	F	7	1.7
AFDK-H5	PRJEB39275	SAMEA7050659	Afar	Dulecha	Kefis	167502638	50	38	47	F	7	1
AFDK-H6	PRJEB39275	SAMEA7050660	Afar	Dulecha	Kefis	195647263	58	45	55	F	8	0.6
AFDK-H7	PRJEB39275	SAMEA7050661	Afar	Dulecha	Kefis	151191455	45	36	43	F	8	1
AFDK-H8	PRJEB39275	SAMEA7050662	Afar	Dulecha	Kefis	165552066	49	40	47	F	12	1.4
AFDK-H9	PRJEB39275	SAMEA7050663	Afar	Dulecha	Kefis	155092561	46	35	44	F		1.2
AFG-1H	PRJEB39275	SAMEA7050664	Amhara	Fagita Lekoma	Gafera	256197086	77	63	72	F	6	1.5
AFG-2C1	PRJEB39275	SAMEA7050665	Amhara	Fagita Lekoma	Gafera	283120181	85	68	80	M	6	1.5
AFG-2H	PRJEB39275	SAMEA7050666	Amhara	Fagita Lekoma	Gafera	265669387	79	65	75	F	6	1.5
AFG-3C	PRJEB39275	SAMEA7050667	Amhara	Fagita Lekoma	Gafera	270092780	81	65	76	M	5	1.5
AFG-3H	PRJEB39275	SAMEA7050668	Amhara	Fagita Lekoma	Gafera	143021468	43	34	40	F	8	1.5
AFG-4H_P_Y	PRJEB39275	SAMEA7050669	Amhara	Fagita Lekoma	Gafera	171882362	51	42	48	F	7	1.5
AFG-5C	PRJEB39275	SAMEA7050670	Amhara	Fagita Lekoma	Gafera	236189159	71	58	67	M	6	1.5
AFG-5H	PRJEB39275	SAMEA7050671	Amhara	Fagita Lekoma	Gafera	280842991	84	68	79	F	4	1
AFG-6H	PRJEB39275	SAMEA7050672	Amhara	Fagita Lekoma	Gafera	194232239	58	47	55	F	4	1
AFG-8C	PRJEB39275	SAMEA7050673	Amhara	Fagita Lekoma	Gafera	276246879	83	67	78	M	5	1
AGT-10C	PRJEB39275	SAMEA7050674	Amhara	Gondar Zuria	Tsion Teguaz	174781192	52	41	49	M	8	1
AGT-1C	PRJEB39275	SAMEA7050675	Amhara	Gondar Zuria	Tsion Teguaz	162035613	48	38	46	M	19	1.5
AGT-2C	PRJEB39275	SAMEA7050676	Amhara	Gondar Zuria	Tsion Teguaz	166589303	50	39	47	M	8	1.5
AGT-3H	PRJEB39275	SAMEA7050677	Amhara	Gondar Zuria	Tsion Teguaz	178769791	53	43	50	F	12	1.5
AGT-4H	PRJEB39275	SAMEA7050678	Amhara	Gondar Zuria	Tsion Teguaz	240100167	72	58	68	F	12	1
AGT-5H	PRJEB39275	SAMEA7050679	Amhara	Gondar Zuria	Tsion Teguaz	164846479	49	39	46	F	12	1
AGT-6H	PRJEB39275	SAMEA7050680	Amhara	Gondar Zuria	Tsion Teguaz	171572055	51	41	48	F	15	1
AGT-7H	PRJEB39275	SAMEA7050681	Amhara	Gondar Zuria	Tsion Teguaz	158099542	47	37	45	F	18	1
AGT-8H	PRJEB39275	SAMEA7050682	Amhara	Gondar Zuria	Tsion Teguaz	160926681	48	38	45	F	9	1
AGT-9C	PRJEB39275	SAMEA7050683	Amhara	Gondar Zuria	Tsion Teguaz	158368711	47	37	45	M	9	1.5
AK025A-10C-059	PRJEB39275	SAMEA7050684	Amhara	Kalu	0-25Adane	221676963	66	52	62	M	6	1.1
AK025A-1HC-55	PRJEB39275	SAMEA7050685	Amhara	Kalu	0-25Adane	214924783	64	52	61	F	12	1.1
AK025A-2H-149	PRJEB39275	SAMEA7050686	Amhara	Kalu	0-25Adane	170277513	51	41	48	F	8	0.88
AK025A-3H-85	PRJEB39275	SAMEA7050687	Amhara	Kalu	0-25Adane	211196221	63	49	59	F	7	1.4
AK025A-4H-070	PRJEB39275	SAMEA7050688	Amhara	Kalu	0-25Adane	171437883	51	41	48	F		1.06
AK025A-5H-71	PRJEB39275	SAMEA7050689	Amhara	Kalu	0-25Adane	154447981	46	37	43	F	6	0.86
AK025A-6H-97	PRJEB39275	SAMEA7050690	Amhara	Kalu	0-25Adane	156387764	47	37	44	F	11	1.14
AK025A-7C1	PRJEB39275	SAMEA7050691	Amhara	Kalu	0-25Adane	164065755	49	38	46	M	8	1.2
AK025A-8C	PRJEB39275	SAMEA7050692	Amhara	Kalu	0-25Adane	238209857	71	56	67	M		
AK025A-8C-50	PRJEB39275	SAMEA7050693	Amhara	Kalu	0-25Adane	171979726	51	42	48	M	8	1.34
AKA-10H-11	PRJEB39275	SAMEA7050694	Amhara	Kalu	Arabo	156126238	47	35	44	F		1.1
AKA-1C	PRJEB39275	SAMEA7050695	Amhara	Kalu	Arabo	161880229	48	38	46	M		0.9
AKA-2H	PRJEB39275	SAMEA7050696	Amhara	Kalu	Arabo	146145065	44	34	41	F	5	0.82
AKA-3C	PRJEB39275	SAMEA7050697	Amhara	Kalu	Arabo	437080246	131	99	123	M	5	1.14
AKA-4H	PRJEB39275	SAMEA7050698	Amhara	Kalu	Arabo	164770789	49	40	46	F	12	1.2
AKA-5H	PRJEB39275	SAMEA7050699	Amhara	Kalu	Arabo	172851099	52	42	49	F	6	0.85
AKA-6H	PRJEB39275	SAMEA7050700	Amhara	Kalu	Arabo	181386777	54	43	51	F	8	1.2
AKA-7C	PRJEB39275	SAMEA7050701	Amhara	Kalu	Arabo	153111811	46	36	43	M	8	1.2
AKA-8H	PRJEB39275	SAMEA7050702	Amhara	Kalu	Arabo	147763907	44	35	42	F	14	1.3
AKA-9C	PRJEB39275	SAMEA7050703	Amhara	Kalu	Arabo	153394599	46	34	43	M	8	1.5
AMAM-10H-108	PRJEB39275	SAMEA7050704	Amhara	Menz Gera Midir	Alfa Midir/05/	219841013	66	53	62	F	7	1
AMAM-1H-031	PRJEB39275	SAMEA7050705	Amhara	Menz Gera Midir	Alfa Midir/05/	185325710	55	44	52	F	24	1.12
AMAM-2C-151	PRJEB39275	SAMEA7050706	Amhara	Menz Gera Midir	Alfa Midir/05/	167703397	50	41	47	M	12	1.01
AMAM-3H-124	PRJEB39275	SAMEA7050707	Amhara	Menz Gera Midir	Alfa Midir/05/	213711633	64	48	60	F	14	0.7
AMAM-4H-115	PRJEB39275	SAMEA7050708	Amhara	Menz Gera Midir	Alfa Midir/05/	174718288	52	39	49	F	8	1.26
AMAM-5C-147	PRJEB39275	SAMEA7050709	Amhara	Menz Gera Midir	Alfa Midir/05/	165835363	50	38	47	M	9	1.38
AMAM-6H-120	PRJEB39275	SAMEA7050710	Amhara	Menz Gera Midir	Alfa Midir/05/	189256702	57	43	53	F	9	1.08
AMAM-7C-116	PRJEB39275	SAMEA7050711	Amhara	Menz Gera Midir	Alfa Midir/05/	221757177	66	53	62	M	6	1.16
AMAM-8H-101	PRJEB39275	SAMEA7050712	Amhara	Menz Gera Midir	Alfa Midir/05/	173924627	52	40	49	F	12	0.78
AMAM-9C-107	PRJEB39275	SAMEA7050713	Amhara	Menz Gera Midir	Alfa Midir/05/	169714035	51	39	48	M	9	1.52
AMNA-10C	PRJEB39275	SAMEA7050714	Amhara	Menz Gera Midir	Negasi Amba/07/	181657517	54	44	51	M	12	1.32
AMNA-1H-102	PRJEB39275	SAMEA7050715	Amhara	Menz Gera Midir	Negasi Amba/07/	208716330	62	50	59	F	12	1.18
AMNA-2C-104	PRJEB39275	SAMEA7050716	Amhara	Menz Gera Midir	Negasi Amba/07/	173300406	52	40	49	M	12	1.2
AMNA-3H-170	PRJEB39275	SAMEA7050717	Amhara	Menz Gera Midir	Negasi Amba/07/	178208155	53	40	50	F	12	1.04
AMNA-4H-121	PRJEB39275	SAMEA7050718	Amhara	Menz Gera Midir	Negasi Amba/07/	170292167	51	40	48	F	12	1.1
AMNA-5C-138	PRJEB39275	SAMEA7050719	Amhara	Menz Gera Midir	Negasi Amba/07/	173866786	52	39	49	M	15	0.98
AMNA-6H-165	PRJEB39275	SAMEA7050720	Amhara	Menz Gera Midir	Negasi Amba/07/	211546437	63	49	60	F	11.5	1
AMNA-7H-144	PRJEB39275	SAMEA7050721	Amhara	Menz Gera Midir	Negasi Amba/07/	154462951	46	37	43	F	9	1.2
AMNA-8H	PRJEB39275	SAMEA7050722	Amhara	Menz Gera Midir	Negasi Amba/07/	203003582	61	49	57	M		
AMNA-9C-171	PRJEB39275	SAMEA7050723	Amhara	Menz Gera Midir	Negasi Amba/07/	172104718	51	42	48	M	12	1.7
ASA-10H	PRJEB39275	SAMEA7050724	Amhara	South Achefer	Ashuda	257638319	77	62	73	F	8	1.5
ASA-1C	PRJEB39275	SAMEA7050725	Amhara	South Achefer	Ashuda	275105168	82	68	77	M	6	1.5
ASA-2H	PRJEB39275	SAMEA7050726	Amhara	South Achefer	Ashuda	171251993	51	40	48	F	8	1
ASA-3H	PRJEB39275	SAMEA7050727	Amhara	South Achefer	Ashuda	186056888	56	45	52	F	8	1
ASA-4C	PRJEB39275	SAMEA7050728	Amhara	South Achefer	Ashuda	240955666	72	58	68	M	5	1.5
ASA-5H	PRJEB39275	SAMEA7050729	Amhara	South Achefer	Ashuda	238719443	71	59	67	F	8	1
ASA-6H	PRJEB39275	SAMEA7050730	Amhara	South Achefer	Ashuda	209755600	63	51	59	F	9	1
ASA-7H	PRJEB39275	SAMEA7050731	Amhara	South Achefer	Ashuda	157731335	47	38	44	F	11	1.5
ASA-8C	PRJEB39275	SAMEA7050732	Amhara	South Achefer	Ashuda	237053984	71	57	67	M	7	1.5
ASA-9C	PRJEB39275	SAMEA7050733	Amhara	South Achefer	Ashuda	196938915	59	48	55	M	6	1.5
ASD-1C	PRJEB39275	SAMEA7050734	Amhara	South Achefer	Dikuli	160638411	48	38	45	M	9	1.5
ASD-1H	PRJEB39275	SAMEA7050735	Amhara	South Achefer	Dikuli	199066825	59	48	56	F	9	1
ASD-2C	PRJEB39275	SAMEA7050736	Amhara	South Achefer	Dikuli	219215794	65	53	62	M	18	2
ASD-2H	PRJEB39275	SAMEA7050737	Amhara	South Achefer	Dikuli	151513421	45	35	43	F	9	1
ASD-3H	PRJEB39275	SAMEA7050738	Amhara	South Achefer	Dikuli	161665189	48	38	46	F	9	1.5
ASD-4H	PRJEB39275	SAMEA7050739	Amhara	South Achefer	Dikuli	159930126	48	37	45	F	10	1
ASD-5C	PRJEB39275	SAMEA7050740	Amhara	South Achefer	Dikuli	255279427	76	62	72	M	12	1
ASD-6H	PRJEB39275	SAMEA7050741	Amhara	South Achefer	Dikuli	285006193	85	69	80	F	12	1.5
ASD-7H	PRJEB39275	SAMEA7050742	Amhara	South Achefer	Dikuli	175380650	52	42	49	F	12	1.5
ASD-8C	PRJEB39275	SAMEA7050743	Amhara	South Achefer	Dikuli	256119589	77	63	72	M	9	1.5
BGDG-10H	PRJEB39275	SAMEA7050744	Gumuz	Dibate	Gesses	207820060	62	49	59	F		
BGDG-1C-080	PRJEB39275	SAMEA7050745	Gumuz	Dibate	Gesses	208589648	62	50	59	M		1.48
BGDG-2C-95	PRJEB39275	SAMEA7050746	Gumuz	Dibate	Gesses	167721494	50	41	47	M		0.92
BGDG-3H-12	PRJEB39275	SAMEA7050747	Gumuz	Dibate	Gesses	168341912	50	39	47	F	8	1
BGDG-4C-51	PRJEB39275	SAMEA7050748	Gumuz	Dibate	Gesses	159377844	48	37	45	M		1.9
BGDG-5H-2	PRJEB39275	SAMEA7050749	Gumuz	Dibate	Gesses	168898025	50	39	48	F	12	1
BGDG-6C	PRJEB39275	SAMEA7050750	Gumuz	Dibate	Gesses	263030959	76	61	74	M		
BGDG-7H-23	PRJEB39275	SAMEA7050751	Gumuz	Dibate	Gesses	181115763	54	42	51	F		1
BGDG-8H	PRJEB39275	SAMEA7050752	Gumuz	Dibate	Gesses	222615507	66	53	63	F	7	1.25
BGDG-9H	PRJEB39275	SAMEA7050753	Gumuz	Dibate	Gesses	175232510	52	42	49	F	9	1.4
BGDK-10C-14	PRJEB39275	SAMEA7050754	Gumuz	Dibate	Kido	192733628	58	47	54	M	6	1.2
BGDK-11H-56	PRJEB39275	SAMEA7050755	Gumuz	Dibate	Kido	181206673	54	44	51	F		0.82
BGDK-12C	PRJEB39275	SAMEA7050756	Gumuz	Dibate	Kido	173198494	52	42	49	M		0.84
BGDK-3C	PRJEB39275	SAMEA7050757	Gumuz	Dibate	Kido	200476766	60	47	56	M		
BGDK-5H-82	PRJEB39275	SAMEA7050758	Gumuz	Dibate	Kido	167630197	50	41	47	F	6	1.2
BGDK-6H	PRJEB39275	SAMEA7050759	Gumuz	Dibate	Kido	206472010	62	47	58	F		
BGDK-7H	PRJEB39275	SAMEA7050760	Gumuz	Dibate	Kido	202670391	60	44	57	F		
BGDK-8H	PRJEB39275	SAMEA7050761	Gumuz	Dibate	Kido	177890612	52	40	50	F		
BGDK-9H	PRJEB39275	SAMEA7050762	Gumuz	Dibate	Kido	172820697	52	42	49	F		0.6
ODB-10H-003	PRJEB39275	SAMEA7050763	Oromia	Dugda	Bekele Girissa	206828761	62	51	58	F	6	1
ODB-1H-076	PRJEB39275	SAMEA7050764	Oromia	Dugda	Bekele Girissa	170679500	51	42	48	F	5	1
ODB-2C-083	PRJEB39275	SAMEA7050765	Oromia	Dugda	Bekele Girissa	180254512	54	42	51	M	6	1
ODB-3H	PRJEB39275	SAMEA7050766	Oromia	Dugda	Bekele Girissa	203341979	61	46	57	F	9	1
ODB-4H-002	PRJEB39275	SAMEA7050767	Oromia	Dugda	Bekele Girissa	184572600	55	45	52	F	12	1.5
ODB-5H	PRJEB39275	SAMEA7050768	Oromia	Dugda	Bekele Girissa	194734764	58	44	55	F	5	1
ODB-6C-093	PRJEB39275	SAMEA7050769	Oromia	Dugda	Bekele Girissa	186013314	56	41	52	M	12	1
ODB-7C	PRJEB39275	SAMEA7050770	Oromia	Dugda	Bekele Girissa	202362491	60	47	57	M	5	1
ODB-8H-013	PRJEB39275	SAMEA7050771	Oromia	Dugda	Bekele Girissa	184211704	55	42	52	F	5	1
ODB-9H	PRJEB39275	SAMEA7050772	Oromia	Dugda	Bekele Girissa	195953021	59	48	55	F	6	1
ODS-10H-068	PRJEB39275	SAMEA7050773	Oromia	Dugda	Shubi Gemo	197892965	59	47	56	F	7	1
ODS-1H	PRJEB39275	SAMEA7050774	Oromia	Dugda	Shubi Gemo	268103297	79	61	75	F		
ODS-2H-88	PRJEB39275	SAMEA7050775	Oromia	Dugda	Shubi Gemo	181845407	54	42	51	F		1
ODS-3C	PRJEB39275	SAMEA7050776	Oromia	Dugda	Shubi Gemo	210036912	63	50	57	M		
ODS-4H-010	PRJEB39275	SAMEA7050777	Oromia	Dugda	Shubi Gemo	207470216	62	49	58	F	5	1
ODS-5C-086	PRJEB39275	SAMEA7050778	Oromia	Dugda	Shubi Gemo	258231231	77	60	73	M	5	1
ODS-6C-098	PRJEB39275	SAMEA7050779	Oromia	Dugda	Shubi Gemo	224707183	67	55	63	M	7	1
ODS-7H	PRJEB39275	SAMEA7050780	Oromia	Dugda	Shubi Gemo	190458745	57	44	54	F		
ODS-8H-042	PRJEB39275	SAMEA7050781	Oromia	Dugda	Shubi Gemo	192840864	58	47	54	F	7	1.5
ODS-9H-016	PRJEB39275	SAMEA7050782	Oromia	Dugda	Shubi Gemo	203753022	61	47	57	F	12	1
SDK-10C-004	PRJEB39275	SAMEA7050783	SNNPR	Dara	Kumato	179621620	54	42	51	M	10	1
SDK-1H-080	PRJEB39275	SAMEA7050784	SNNPR	Dara	Kumato	191383859	57	44	54	F	12	
SDK-2C	PRJEB39275	SAMEA7050785	SNNPR	Dara	Kumato	210686761	63	49	59	M	18	1.7
SDK-3H-054	PRJEB39275	SAMEA7050786	SNNPR	Dara	Kumato	209572541	63	50	59	F	18	1.7
SDK-4C-062	PRJEB39275	SAMEA7050787	SNNPR	Dara	Kumato	223374021	67	55	63	M	12	1.5
SDK-5H	PRJEB39275	SAMEA7050788	SNNPR	Dara	Kumato	191841797	56	43	54	F		
SDK-6H-052	PRJEB39275	SAMEA7050789	SNNPR	Dara	Kumato	187517459	56	46	53	F	12	1.5
SDK-7H	PRJEB39275	SAMEA7050790	SNNPR	Dara	Kumato	205742068	61	48	58	F	12	1.6
SDK-8C-021	PRJEB39275	SAMEA7050791	SNNPR	Dara	Kumato	210263073	63	49	59	M	12	1.6
SDK-9H-026	PRJEB39275	SAMEA7050792	SNNPR	Dara	Kumato	190512885	57	47	54	F	10	1
SDL-10H-017	PRJEB39275	SAMEA7050793	SNNPR	Dara	Loya	218242387	65	53	61	F	7	1.5
SDL-1C-091	PRJEB39275	SAMEA7050794	SNNPR	Dara	Loya	494126576	148	123	139	M	12	1.5
SDL-2H-064	PRJEB39275	SAMEA7050795	SNNPR	Dara	Loya	167570521	50	41	47	F	7	1.5
SDL-3C-019	PRJEB39275	SAMEA7050796	SNNPR	Dara	Loya	209340893	63	51	59	M	8	2
SDL-4H-066	PRJEB39275	SAMEA7050797	SNNPR	Dara	Loya	209633949	63	48	59	F	12	1
SDL-5H-008	PRJEB39275	SAMEA7050798	SNNPR	Dara	Loya	195060857	58	46	55	F	7	1
SDL-6H-057	PRJEB39275	SAMEA7050799	SNNPR	Dara	Loya	187214881	56	44	53	F	8	1
SDL-7C-035	PRJEB39275	SAMEA7050800	SNNPR	Dara	Loya	220419363	66	52	62	F	9	1.5
SDL-8C-090	PRJEB39275	SAMEA7050801	SNNPR	Dara	Loya	173775632	52	41	49	M	7	1
SDL-9H-013	PRJEB39275	SAMEA7050802	SNNPR	Dara	Loya	215730805	64	52	61	F	8	1.5
TGENF_110	PRJEB39275	SAMEA7050803	Tigray	Enderta	Meseret	256139496	74	56	72	F	25	1.25
TGENF_128	PRJEB39275	SAMEA7050804	Tigray	Enderta	Meseret	165845435	48	37	47	F	26	0.8
TGENF_134	PRJEB39275	SAMEA7050805	Tigray	Enderta	Meseret	203215770	61	48	57	F	25	1
TGENF_145	PRJEB39275	SAMEA7050806	Tigray	Enderta	Meseret	334654947	99	77	94	F	26	1.3
TGENF_177	PRJEB39275	SAMEA7050807	Tigray	Enderta	Meseret	158159539	46	35	45	F	28	0.85
TGENF_178	PRJEB39275	SAMEA7050808	Tigray	Enderta	Meseret	251142145	73	58	71	F	30	1.7
TGENM_135	PRJEB39275	SAMEA7050809	Tigray	Enderta	Meseret	177253135	53	41	50	M	28	1.55
TGENM_146	PRJEB39275	SAMEA7050810	Tigray	Enderta	Meseret	194712607	58	44	55	M	30	1.8
TGENM_150	PRJEB39275	SAMEA7050811	Tigray	Enderta	Meseret	199736461	60	45	56	M	26	1.45
TGENM_175	PRJEB39275	SAMEA7050812	Tigray	Enderta	Meseret	215840911	63	49	61	M	26	1.2
TMLHA_13H	PRJEB39275	SAMEA7050813	Tigray	Merebleke	Hadush Adi	171655146	51	42	48	F	8	1.2
TMLHA-04C	PRJEB39275	SAMEA7050814	Tigray	Merebleke	Hadush Adi	159294195	48	39	45	M		2.6
TMLHA-19H	PRJEB39275	SAMEA7050815	Tigray	Merebleke	Hadush Adi	248627376	74	58	70	F		1.2
TMLHA-54H	PRJEB39275	SAMEA7050816	Tigray	Merebleke	Hadush Adi	198560606	58	45	56	F		
TMLHA-66C	PRJEB39275	SAMEA7050817	Tigray	Merebleke	Hadush Adi	456440319	136	107	129	M		1.83
TMLHA-86H	PRJEB39275	SAMEA7050818	Tigray	Merebleke	Hadush Adi	157424722	47	36	44	F	12	1.3
TMLHA-90C	PRJEB39275	SAMEA7050819	Tigray	Merebleke	Hadush Adi	197521255	58	44	56	M		
TMLHA-91C	PRJEB39275	SAMEA7050820	Tigray	Merebleke	Hadush Adi	166424117	50	37	47	F		1
TMLHA-96H	PRJEB39275	SAMEA7050821	Tigray	Merebleke	Hadush Adi	156203276	47	36	44	F	10	1.43
TMLM-_C94	PRJEB39275	SAMEA7050822	Tigray	Merebleke	Mihquan	162650188	49	38	46	M	12	1.45
TMLM-26C	PRJEB39275	SAMEA7050823	Tigray	Merebleke	Mihquan	177855728	52	39	50	M		
TMLM-6H	PRJEB39275	SAMEA7050824	Tigray	Merebleke	Mihquan	166357341	50	41	47	F	12	1.1
TMLM-C30	PRJEB39275	SAMEA7050825	Tigray	Merebleke	Mihquan	148859908	44	36	42	M	12	2.1
TMLM-C89	PRJEB39275	SAMEA7050826	Tigray	Merebleke	Mihquan	186763080	56	45	53	M	10	1.1
TMLM-H18	PRJEB39275	SAMEA7050827	Tigray	Merebleke	Mihquan	174320520	52	42	49	F	12	1.65
TMLM-H53	PRJEB39275	SAMEA7050828	Tigray	Merebleke	Mihquan	180324068	54	43	51	F	10	1.6
TMLM-H57	PRJEB39275	SAMEA7050829	Tigray	Merebleke	Mihquan	145397265	43	33	41	F	10	1.1
TMLM-H72	PRJEB39275	SAMEA7050830	Tigray	Merebleke	Mihquan	165668541	50	38	47	F	12	1.3
TMLM-H84	PRJEB39275	SAMEA7050831	Tigray	Merebleke	Mihquan	169294406	51	39	48	F	12	1.57
TSSG-07H	PRJEB39275	SAMEA7050832	Tigray	Sahareti Samire	Gijet	182631706	55	43	51	F		
TSSG-29H	PRJEB39275	SAMEA7050833	Tigray	Sahareti Samire	Gijet	150096758	45	37	42	F	15	1.7
TSSG-37C	PRJEB39275	SAMEA7050834	Tigray	Sahareti Samire	Gijet	227048103	68	52	64	M	9	1.5
TSSG-40H	PRJEB39275	SAMEA7050835	Tigray	Sahareti Samire	Gijet	142314691	43	34	40	F	7	0.9
TSSG-43C	PRJEB39275	SAMEA7050836	Tigray	Sahareti Samire	Gijet	174185407	52	42	49	M	15	1.2
TSSG-44C	PRJEB39275	SAMEA7050837	Tigray	Sahareti Samire	Gijet	447119431	134	103	126	M	6	1.4
TSSG-47H	PRJEB39275	SAMEA7050838	Tigray	Sahareti Samire	Gijet	157567523	47	36	44	F	12	1.4
TSSG-48H	PRJEB39275	SAMEA7050839	Tigray	Sahareti Samire	Gijet	319244953	94	75	90	F		
TSSG-49C	PRJEB39275	SAMEA7050840	Tigray	Sahareti Samire	Gijet	167635858	50	39	47	M	5	1.7
TSSM-17C	PRJEB39275	SAMEA7050841	Tigray	Sahareti Samire	Metkilimat	162493989	49	39	46	M	6	1.2
TSSM-21H	PRJEB39275	SAMEA7050842	Tigray	Sahareti Samire	Metkilimat	157876144	47	36	44	F	6	0.9
TSSM-35H	PRJEB39275	SAMEA7050843	Tigray	Sahareti Samire	Metkilimat	158631341	47	39	45	F	7	1.6
TSSM-52C	PRJEB39275	SAMEA7050844	Tigray	Sahareti Samire	Metkilimat	163067895	49	38	46	M	8	1.5
TSSM-62H	PRJEB39275	SAMEA7050845	Tigray	Sahareti Samire	Metkilimat	172603563	52	42	49	F	6	1.3
TSSM-64H	PRJEB39275	SAMEA7050846	Tigray	Sahareti Samire	Metkilimat	192284955	57	46	54	F	12	1.2
TSSM-68C	PRJEB39275	SAMEA7050847	Tigray	Sahareti Samire	Metkilimat	156208128	47	38	44	M	6	1.7
TSSM-81H	PRJEB39275	SAMEA7050848	Tigray	Sahareti Samire	Metkilimat	158955463	47	36	45	F	8	1.6
TSSM-92H	PRJEB39275	SAMEA7050849	Tigray	Sahareti Samire	Metkilimat	175441722	52	41	49	F	10	1.2
TSSM-99C	PRJEB39275	SAMEA7050850	Tigray	Sahareti Samire	Metkilimat	190253350	57	43	54	M	8	1.3
